# An Examination of Correlates of Quality of Life in Children and Youth With Mental Health Issues

**DOI:** 10.3389/fpsyt.2021.709516

**Published:** 2021-09-01

**Authors:** Angela Celebre, Shannon L. Stewart, Laura Theall, Natalia Lapshina

**Affiliations:** ^1^Faculty of Education, Western University, London, ON, Canada; ^2^Child and Parent Resource Institute, London, ON, Canada

**Keywords:** children and youth, mental health, quality of life, mental state indicators, interRAI

## Abstract

Quality of life (QoL) is significantly lower in children with mental health issues compared to those who are typically developing or have physical health problems. However, little research has examined factors associated with QoL in this particularly vulnerable population. To address this limitation, 347 clinically referred children and adolescents were assessed using the interRAI Child and Youth Mental Health (ChYMH) Assessment and Self-reported Quality of Life- Child and Youth Mental Health (QoL-ChYMH). Hierarchical multiple linear regression analyses were conducted to examine QoL at the domain-specific level. Children and adolescents who experienced heightened anhedonia and depressive symptoms reported lower social QoL (e.g., family, friends and activities; *p* = 0.024, 0.046, respectively). Additionally, children and youth who experienced heightened depressive symptoms reported lower QoL at the individual level (e.g., autonomy, health; *p* = 0.000), and level of basic needs (e.g., food, safety; *p* = 0.013). In contrast, no mental state indicators were associated with QoL related to services (e.g., school, treatment). Due to the paucity of research examining predictors of QoL in children and youth with mental health challenges, this study contributes to the field in assisting service providers with care planning and further providing implications for practice.

## Introduction

Untreated mental health issues can have an adverse impact on both individuals and society. Notably, individuals with mental health issues have a significantly lower quality of life (QoL) compared to the general population, as well as those with physical health problems (1–4). While a vast amount of research has investigated determinants of QoL in adults with mental illness, including sociodemographic data, symptom severity, functionality, personality factors, and social interactions [e.g., (5–7)], the same is unfortunately not true for the pediatric population. To address this gap in the literature, the present study examined whether certain factors (i.e., mental state indicators) were associated with QoL in children and youth receiving mental health services.

### Quality of Life

Although there is no general consensus on the definition of quality of life, it is agreed that QoL represents a multi-dimensional construct that integrates a number of different domains, such as psychological, physical, and social well-being. There has been an increase in the development and use of QoL tools in recent decades, which has coincided with a shift within the healthcare system towards recognizing the importance of the client's preferences and life experiences in addition to their symptomology [e.g., (8–14)]. These tools are being used for a variety of purposes, such as to evaluate treatment outcomes, to help predict physical and mental health problems, and to facilitate shared clinical decision-making between clinicians and patients (15–18). There is also increased awareness around the value in obtaining self-reports of QoL from children and adolescents in particular, due in large part to the parent-proxy problem. Specifically, extensive research has shown poor agreement between parent-proxy and child reports of QoL (19–21). Therefore, it is strongly advised for children to report on their own QoL whenever possible.

### Quality of Life Measurement

QoL has been conceptualized in a number of different ways in the literature because it is a latent construct. For example, researchers have proposed four main approaches to QoL, including objective measures, subjective measures, health-related quality of life (HR-QOL), and health-economic measures (22). Subjective and HR-QOL measures represent two of the most common approaches to QoL measurement in child and adolescent mental health research. Subjective measures are largely focused on the individual's perceptions, such as a sense of well-being and happiness (23). HR-QOL measures have been developed as a result of the healthcare field realizing that traditional measures of disease do not fully capture the effects of illness (24). While the broad domains among most QoL tools are fairly consistent, the specific sub-domains vary depending on the particular measure. This variability in structure can make it quite difficult to compare QoL measures (25).

While there has been an increase in the development of QoL instruments for children and youth, the majority of these instruments are intended for pediatric chronic care [e.g., (26)]. However, in response to the need for more diverse QoL tools, interRAI has developed several self-report QoL measures (11), including those specifically for children and youth receiving mental health services (13). While there is some overlap among these surveys, it was critical to develop a tool specifically for young persons because there are different considerations when assessing QoL in a pediatric population, such as the influence of family relationships and the school environment. A common approach to assessing QoL across the lifespan fosters engagement and self-determination in the treatment process, providing opportunities for improved outcomes while enhancing service system integration.

The Self-reported Quality of Life- Child and Youth Mental Health (QoL-ChYMH) is one of the newest additions to the interRAI Child and Youth suite of instruments (13). The tool assesses the perception of well-being and life satisfaction of children and youth with mental health issues. Importantly, the QoL-ChYMH provides these young persons with a voice, promoting engagement in their own mental healthcare. The purpose of this self-report tool has been to assist healthcare professionals in identifying a young person's strengths and needs in order to maximize treatment gains while improving QoL.

### Quality of Life and Mental Health

The relationship between mental health issues and lower QoL is well-supported in the adult literature. For example, research has shown that QoL is reduced in those who have been diagnosed with various psychiatric disorders, such as depression, anxiety, bipolar disorder, schizophrenia, and others [e.g., (27–30)]. With respect to schizophrenia, studies have found that negative symptoms have a significant inverse correlation to QoL; however, the relationship between positive symptoms and QoL is less clear [e.g., (31, 32)]. One study compared QoL among different mental health disorders, including mood, anxiety, somatoform, alcohol, and eating disorders, and found that mood disorders accounted for significantly more impairment across all HR-QOL domains, whereas other disorders only affected certain domains (33). Importantly, researchers have found that even subthreshold symptom levels are associated with lower QoL. This suggests that an individual does not need to receive a psychiatric diagnosis in order for their QoL to be diminished.

QoL in childhood mental health disorders is much less established compared to other fields, including various childhood somatic diseases as well as adult mental health (34). However, Jonsson et al. (35) conducted a recent review examining the impact of childhood mental health disorders on QoL. Overall, they found that clinical populations reported lower QoL compared to healthy control groups. Further, the authors noted that studies for large diagnostic groups, such as anxiety disorders, depressive disorders, and (early onset) schizophrenia are largely lacking. Within the child and youth literature, research has primarily focused on the relationship between QoL and a particular mental health condition as opposed to comparisons between conditions. For example, lower QoL has been reported in young persons with depressive symptoms (36), anxiety symptoms (37), and ADHD (particularly when measured via proxy-report as opposed to self-report) (20).

## Current Study

While there is a growing awareness of the importance of QoL within the healthcare field, the literature examining the association between mental health and QoL in children and youth is in its infancy. The purpose of this study was to address the current gaps in the literature, given that there is a paucity of research: (1) examining the relationship between the level of impairment/symptom severity and QoL, (2) comparing mental health indicators rather than disorders, and (3) examining QoL at the domain-specific as opposed to overall level. To address these gaps, the present study examined the association between the severity level of various mental state indicators (i.e., depressive symptoms, anxiety, hyperactivity/distractibility, positive symptoms, and anhedonia) and domain-specific QoL, among a sample of children and youth referred for mental health care. On the basis of previous literature, it was predicted that depressive symptoms would be significantly associated with the greatest number of QoL sub-domains. However, analyses examining the predictive effect of the remaining mental state indicators were exploratory by nature since this is the first study, to our knowledge, to include such a breadth of clinical factors in the same multivariate model.

## Method

### Sample

A convenience sample of 347 clinically referred English-speaking children and youth who have accessed services from a tertiary center for young persons with complex needs (including autism, developmental disabilities, and mental health) in the province of Ontario over a 3-year period participated in this study. All children and youth were referred for services through their family physicians, pediatricians, school personnel, or other allied health care professionals.

The interRAI Child and Youth Mental Health Assessment (ChYMH) (38) and QoL-ChYMH (13) were administered as part of typical clinical practice to each child or youth upon accessing mental health services at the supporting agency. Both males (72.3%) and females (27.7%) ranging in age from 7 to 18 years old (M = 10.89, SD = 2.76) were included in this study. Inpatient (25.9%) and outpatient (74.1%) children and youth were included in the current study. Additional selected characteristics of the sample population are included in Table 1.

**Table 1:** Selected characteristics of sample population.

**Characteristic**	**% of Sample**
**Marital status of parents**	
Never married	18.8%
Married	49.7%
Partner or significant other	3.2%
Separated	9.8%
Divorced	12.1%
Marital status unknown	6.1%
**Guardianship**	
Both parents	59.7%
Mother only	24.8%
Father only	2.9%
Neither parent, but other relative(s) or non-relative(s)	8.6%
Child protection agency	3.7%
**History of foster family placement**	
None	80.6%
One foster family	10.4%
Multiple foster families	9.0%
**Top diagnoses**	
Attention deficit hyperactivity disorder	58.8%
Anxiety disorders	38.6%
Disruptive behavior disorder	29.4%
Learning or communication disorder	25.6%
Autism spectrum disorder	15.3%

### Procedure

Trained child and youth mental health care service providers (e.g., social workers, child and youth workers, psychologists, psychiatrists, occupational therapists) who completed ChYMH assessments had a diploma or degree in the mental health field, at least 2 years of experience with children and youth, and completed a 2.5-day training program with respect to the administration of the interRAI ChYMH. Completion of the ChYMH assessment took ~60–90 min depending on case complexity. All possible sources were utilized to complete the assessment including information from child/youth and family, medical records, school records, and other collateral documents.

The child or youth accessing mental health services completed the QoL-ChYMH at the same time the assessor completed the ChYMH assessment. The survey takes about 15 min to complete and is suitable for children and youth with a literacy level of grade 2. While there are three versions of the QoL-ChYMH available (i.e., Pre-Service, Post-Service Outpatient, and Post-Service In-patient), this study only used the Pre-Service version.

All data was gathered over a three-year period. Items from the interRAI ChYMH and QoL-ChYMH were included in the current study to investigate the relationship between mental state indicators and QoL in children and adolescents. Assessment information was recorded utilizing a secure online software system that required the entered responses to conform to acceptable values, and subsequently signed as complete.

The present study was approved by Western University's Ethics Board. The data obtained from participants was stored on the interRAI Canada secure server at a partner university. No personal identifiers were obtained or stored on this secure server since each participant is assigned a study-specific participant ID number.

### Measures

#### Self-Reported Quality of Life for Children and Youth Mental Health

The QoL-ChYMH (13) is a self-report survey that assesses the subjective well-being and satisfaction of children and youth 7–18 years, who are receiving services from mental health agencies, hospitals, crisis units, schools, youth justice facilities, and other community services. The 33-item questionnaire was developed based on protective factors and indicators of positive mental health well-established in the literature. The structure of the QoL instrument is composed of four major domains: (1) basic needs (living conditions, food, safety/privacy); (2) social (friends and activities, respect from others, family); (3) individual (autonomy, health); and (4) services (school, treatment). Children and youth are asked to rate how true each statement is for them based on a three-point scale. The QoL-ChYMH has been recommended as “Leading Practice” by Accreditation Canada.

#### interRAI Child and Youth Mental Health Instrument

The interRAI ChYMH (38) is a comprehensive mental health needs assessment. It is comprised of approximately 400 clinical elements that are used to assess medical, psychological, functional, social, and environmental issues for children between 4 and 18 years of age. The instrument is based on a clinician-rated, semi-structured interview format, and completed using all available sources of information, such as direct contact with the child/youth and the family, other service providers (e.g., educators and healthcare professionals), and reviewing previous records (e.g., school records and assessment reports). A number of scales and algorithms are embedded in the instrument that can be used for various applications, including outcome measurement, care planning, resource allocation, and quality improvement. Furthermore, the ChYMH also contains collaborative action plans (CAPs), which are evidence-informed care planning guidelines that can be used to support clinical decision-making (39). These CAPs are triggered based on areas of risk identified through the assessment [e.g., (40)].

Rigorous reliability and validity studies have demonstrated strong psychometric properties across the family of assessment tools targeting various populations, including adults (41–46), as well as children and youth (47–60). For example, one study examined the inter-item reliability of a number of the scales embedded in the ChYMH and several other tools within the interRAI child/youth suite, such as the Anxiety Scale, Aggressive/Disruptive Behavior Scale, Peer Conflict Scale, and Caregiver Distress Scale. The results showed that the scales had strong internal consistency with Cronbach's alpha higher than 0.70 (57).

### Instrument Domains From the QoL-ChYMH

Domain-specific QoL was measured using the QoL-ChYMH, which measures the level of endorsement by the child or youth on a number of items related to the four QoL sub-domains (i.e., basic needs, social, individual, and services). Examples of these items include, “I have choices in how to spend my time”, “I have enough to eat”, and “I get along with other kids”. Each item is rated on a scale from 0 to 2 (0 = *Never true*, 1 = *Sometimes true*, and 2 = *Very or often true*). Four domain-specific scores were calculated for each child or youth by summing the number of items on the QoL-ChYMH pertaining to each of the four domains. The four scores reflect quality of life in relation to basic needs (eight items, scores range from 0 to 16), social (13 items, scores range from 0 to 26), individual (seven items, scores range from 0 to 14), and services (five items, scores range from 0 to 10). Higher scores are indicative of higher quality of life within that domain. Raw scores were converted to z-scores for the purpose of comparative analyses.

### Scales Obtained From the interRAI ChYMH

#### Depressive Symptoms

Depressive symptoms were measured using the Depressive Severity Index (DSI) (50), which measures the frequency and severity of indicators of depression, such as self-deprecation, negative statements, feelings of hopelessness, and sad facial expressions. DSI scores were determined by summing five items, which were rated on a scale from 0 to 4 (0 = *Not present*, to 4 = *Exhibited daily in last 3 days, 3 or more episodes or continuously*). Scores of “4” were subsequently recoded to “3”; thus, scores on the DSI range from 0 to 15 where higher scores are indicative of more severe depressive symptoms. The scale was found to have acceptable reliability, *r* = 0.78.

#### Anxiety Symptoms

Anxiety symptoms were measured using the Anxiety Scale (55), which measures the frequency of the symptoms of anxiety, such as episodes of panic, unrealistic fears, obsessive thoughts, and nightmares. Anxiety scores were determined by summing seven items, which were rated on a scale from 0 to 4 (0 = *Not present*, to 4 = *Exhibited daily in last 3 days, 3 or more episodes or continuously*). Scores on the Anxiety Scale range from 0 to 28 where higher scores are indicative of more severe anxiety symptoms. The scale was found to have acceptable reliability, *r* = 0.68.

#### Hyperactivity and Distractibility

Hyperactive and distractive behavior was measured using the Hyperactivity-Distractibility Scale (47), which calculates the frequency of hyperactivity and distractibility, such as having difficulty paying attention, having an excessive level of activity, and being impulsive. Hyperactivity and distractibility scores were determined by summing four items rated on a scale from 0 to 4 (0 = *Not present*, to 4 = *Exhibited daily in last 3 days, 3 or more episodes or continuously*). Scores on the Hyperactivity-Distractibility Scale range from 0 to 16 where higher scores indicate greater frequency and diversity of disruptive behaviors. The scale was found to have acceptable reliability, *r* = 0.76.

#### Positive Symptoms

Positive symptoms were measured using the Positive Symptoms Scale (61), which calculates the frequency of positive symptoms of psychosis, such as abnormal thought processes, delusions, and hallucinations. Positive symptoms scores were determined by summing four items rated on a scale from 0 to 4 (0 = *Not present*, to 4 = *Exhibited daily in last 3 days, 3 or more episodes or continuously*). Scores of “4” were subsequently recoded to “3”; thus, scores on the Positive Symptoms Scale range from 0 to 12 where higher scores indicate higher levels of positive symptoms. The scale was found to have acceptable reliability, *r* = 0.73.

#### Anhedonia Symptoms

Anhedonia symptoms were measured using the Social Disengagement Scale (57), which assesses the frequency of symptoms related to anhedonia, such as lack of motivation, lack of interest in social interaction, and expressions of a lack of pleasure in life. Anhedonia scores were determined by summing four items rated on a scale from 0 to 4 (0 = *Not present*, to 4 = *Exhibited daily in last 3 days, 3 or more episodes or continuously*). Scores on the Social Disengagement Scale range from 0 to 16 where higher scores indicate higher levels of anhedonia. The scale was found to have acceptable reliability, *r* = 0.66.

### Plan for Analysis

First, frequency and descriptive analyses were conducted for all variables. Second, Spearman's correlation and Mann–Whitney *U* tests were conducted, as appropriate, to assess bivariate relationships between each predictor or control variable and domain-specific QoL. Next, the association between each domain of quality of life (basic needs, social, individual, and services), and predictor variables (depressive symptoms, anxiety, hyperactivity/distractibility, positive symptoms, and anhedonia–controlling for age, sex, and patient status) was examined using hierarchical stepwise multiple linear regression analyses. The first step in each of the analyses included the controlled variables- sex, age, and patient status. The second step included the predictor variables of interest, namely the five mental state indicators- depressive symptoms, anxiety, hyperactivity/distractibility, positive symptoms, and anhedonia. Assumptions testing were conducted for each analysis to control for threats to statistical conclusions, and all analyses were performed using SPSS Statistics Version 25 software (SPSS Inc., Chicago, IL, USA).

Prior to running the analyses, several univariate outliers were detected for each of the five dependent variables (−3.29 > *z* > 3.29, *p* < 0.001). These outliers were subsequently trimmed to z-scores within the acceptable range, and analyses were run with both the original and adjusted data. The outcomes showed no differences, and so it was decided to report results of the analyses including outliers. Furthermore, not all variables were normally distributed; however, bootstrapping did not improve normality, and so the original distributions were used.

## Results

### Preliminary Analyses

The average scores for all dependent and predictor variables are summarized in Table 2. Mann–Whitney *U* tests were employed to examine sex differences in domain-specific QoL. These tests revealed no statistically significant differences between males and females, except on the individual QoL sub-domain, *U*(293) = 10,125.50, *p* = 0.012. Here, males reported significantly higher individual QoL scores compared to females. Spearman's *r* was employed to examine the bivariate relationship between age and QoL; no significant correlations between age and basic needs, social, and services QoL were found. However, a significant negative correlation was found between age and individual QoL (*r*_*s*_ = −0.169, *p* = 0.004). Finally, Mann–Whitney *U* tests were also used to examine differences in QoL scores based on patient status, namely inpatient and outpatient. Here, no differences were found between inpatients and outpatients for the individual or services QoL sub-domains; however, statistically significant differences were found for basic needs [*U*(335) = 8,875.00, *p* = 0.036], and social [*U*(321) = 8,361.50, *p* = 0.037] QoL sub-domains. More specifically, outpatients reported significantly higher QoL scores pertaining to their basic needs (i.e., food and safety) and social relationships (i.e., friends and family) compared to inpatients.

**Table 2:** Summary of mean scores for dependent and predictor variables.

**Variables**	***M (SD)***
**Quality of Life**	
Basic needs	13.36 (2.39)
Social	19.44 (4.05)
Individual	9.88 (2.87)
Services	7.06 (2.19)
**Mental state indicators**	
Depressive severity index	6.05 (4.05)
Anxiety scale	7.50 (5.32)
Hyperactivity-distractibility scale	10.42 (4.63)
Social disengagement scale	3.51 (3.55)
Positive symptoms scale	0.90 (1.93)

### Primary Analyses

All of the following regression analyses were conducted after controlling for age, sex, and patient status, and included the same five predictor variables, namely depressive symptoms, anxiety symptoms, anhedonia, hyperactivity/distractibility, and positive symptoms. Four hierarchical stepwise multiple linear regression analyses were performed to predict domain-specific QoL, namely basic needs, social, individual, and services. In the regression analysis for basic needs QoL, depressive symptoms was the only factor that made a significant contribution, and thus was included in the final model. Specifically, higher levels of depressive symptoms were found to be associated with lower self-reported basic needs QoL (β = −0.143, *t* = −2.509, *p* = 0.013), suggesting that children and youth who experienced heightened depressive symptoms reported less satisfaction with their living conditions, food, and safety/privacy. The final model explained 2.9% of the variance (*p* = 0.011). Table 3 presents the results for the model including the regression coefficients, t-statistics, *p*-values, 95% confidence intervals, and R-squared values (i.e., model fit).

**Table 3:** Regression analysis: basic needs quality of life.

**Model**	***B (SE)***	**β**	***t***	***p***	***95% CI for B***	***R^**2**^***	***ΔR ^**2**^***	***p***
Step 1						0.012	0.022	0.081
Sex	−0.020 (0.126)	−0.009	−0.156	−0.876	[−0.267, 0.228]			
Age	0.029 (0.020)	0.083	1.444	0.150	[−0.011, 0.070]			
Patient status	−0.316 (0.135)	−0.134	−2.337	−0.020	[−0.583, −0.050]			
Step 2						0.029	0.020	0.011
Sex	−0.026 (0.125)	−0.012	−0.209	0.835	[−0.272, 0.220]			
Age	0.033 (0.020)	0.092	1.608	0.109	[−0.007, 0.073]			
Patient status	−0.273 (0.135)	−0.116	−2.017	0.045	[−0.539, −0.007]			
Depressive symptoms	−0.036 (0.014)	−0.143	−2.509	0.013	[−0.065, −0.008]			

In the regression analysis for social QoL, anhedonia and depressive symptoms both made a significant contribution, and thus were included in the final model. Specifically, higher levels of anhedonia (β = −0.145, *t* = −2.274, *p* = 0.024) and depressive symptoms (β = −0.122, *t* = −2.006, *p* = 0.046) were associated with lower self-reported social QoL. This suggests that children and youth who experienced heightened anhedonia and depressive symptoms reported less satisfaction in their relationships with friends and family, in their extra-curricular activities, and generally felt that others did not respect them as much. The final model explained 5.6% of the variance (*p* = 0.001). Table 4 presents the results for the model including the regression coefficients, *t*-statistics, *p*-values, 95% confidence intervals, and R-squared values (i.e., model fit).

**Table 4:** Regression analysis: social quality of life.

**Model**	***B (SE)***	**β**	***t***	***p***	***95% CI for B***	***R^**2**^***	***ΔR^**2**^***	***p***
Step 1						0.017	0.027	0.046
Sex	0.129 (0.122)	0.062	1.054	0.293	[−0.112, 0.369]			
Age	−0.036 (0.020)	−0.107	−1.801	0.073	[−0.075, 0.003]			
Patient status	−0.201 (0.132)	−0.090	−1.527	0.128	[−0.460, 0.058]			
Step 2						0.046	0.031	0.002
Sex	0.110 (0.120)	0.053	0.916	0.361	[−0.127, 0.347]			
Age	−0.016 (0.021)	−0.049	−0.795	0.427	[−0.057, 0.024]			
Patient status	−0.223 (0.130)	−0.100	−1.716	0.087	[−0.479, 0.033]			
Anhedonia	−0.051 (0.016)	−0.186	−3.082	0.002	[−0.083, −0.018]			
Step 3						0.056	0.013	0.001
Sex	0.107 (0.120)	0.051	0.892	0.373	[−0.129, 0.343]			
Age	−0.018 (0.021)	−0.054	−0.889	0.375	[−0.059, 0.022]			
Patient status	−0.187 (0.131)	−0.084	−1.435	0.152	[−0.444, 0.070]			
Anhedonia	−0.039 (0.017)	−0.145	−2.274	0.024	[−0.073, −0.005]			
Depressive symptoms	−0.030 (0.015)	−0.122	−2.006	0.046	[−0.059, −0.001]			

In the regression analysis for individual QoL, depressive symptoms was again the only factor that made a significant contribution, and thus was included in the final model. Specifically, higher levels of depressive symptoms were found to be associated with lower self-reported individual QoL (β = −0.302, *t* = −5.240, *p* = 0.000), indicating that children and youth who experienced heightened depressive symptoms reported less satisfaction with their autonomy and general health. The final model explained 13.9% of the variance (*p* = 0.000). Table 5 presents the results for the model including the regression coefficients, *t*-statistics, *p*-values, 95% confidence intervals, and R-squared values (i.e., model fit).

**Table 5:** Regression analysis: individual quality of life.

**Model**	***B (SE)***	**β**	***t***	***p***	***95% CI for B***	***R^**2**^***	***ΔR^**2**^***	***p***
Step 1						0.051	0.062	0.001
Sex	0.301 (0.129)	0.141	2.329	0.021	[0.046, 0.555]			
Age	−0.064 (0.021)	−0.187	−3.065	0.002	[−0.105, −0.023]			
Patient status	−0.063 (0.135)	−0.028	−0.467	0.641	[−0.328, 0.202]			
Step 2						0.139	0.090	0.000
Sex	0.268 (0.123)	0.126	2.181	0.030	[0.026, 0.511]			
Age	−0.063 (0.020)	−0.184	−3.167	0.002	[−0.102, −0.024]			
Patient status	−0.003 (0.129)	−0.001	−0.022	0.982	[−0.256, 0.251]			
Depressive symptoms	−0.075 (0.014)	−0.302	−5.240	0.000	[−0.103, −0.047]			

Lastly, in the regression analysis for services QoL, none of the factors made a significant contribution, and so no mental state indicators were included in the final model. Table 6 presents the results for the model including the regression coefficients, *t*-statistics, *p*-values, 95% confidence intervals, and R-squared values (i.e., model fit).

**Table 6:** Regression analysis: services quality of life.

**Model**	***B (SE)***	**β**	***t***	***p***	***95% CI for B***	***R^**2**^***	***ΔR^**2**^***	***p***
Step 1						0.013	0.025	0.110
Sex	−0.177 (0.141)	−0.081	−1.257	0.210	[−0.454, 0.100]			
Age	0.009 (0.022)	0.026	0.410	0.682	[−0.034, 0.053]			
Patient status	0.302 (0.148)	0.131	2.045	0.042	[0.011, 0.592]			

## Discussion

While the effect of mental health conditions on QoL has been examined extensively in adult populations, research exploring the relationship between mental health and QoL in pediatric populations is largely lacking. The present study addressed this gap in the literature by examining the association between numerous mental state indicators, namely depressive symptoms, anxiety symptoms, anhedonia, hyperactivity/distractibility, and positive symptoms, and self-reported QoL in children and youth receiving mental health services.

Despite the paucity of extant research examining the relationship between mental health issues and QoL, findings reported herein are consistent with results from the PedsQL, which assessed self-reported QoL in youth with psychiatric disorders. Specifically, the average overall QoL score in the present study was 75.3% compared to 73.3% in the prior study (62). Similar to previous literature, the present study found relatively weak or non-existent relationships between demographic variables and QoL. For example, both sex and age were only weakly correlated with QoL, as no significant differences were found except in the area of individual QoL. Here, males and younger children reported higher QoL scores in this sub-domain than females and older children. Consistent with previous research, boys often report higher QoL compared to girls (particularly within the psychological and physical QoL domains) (63), and younger children (i.e., ages 8–11 years-old) report higher QoL compared to older children (i.e., ages 12–18 years-old) (64).

With respect to the association between patient status (i.e., inpatient vs. outpatient) and QoL, the current study found a significant relationship between patient status and two of the four QoL sub-domains. More specifically, inpatients reported lower social and basic needs QoL compared to outpatients. Of note, because the present study used the pre-service version of the QoL-ChYMH, the inpatient clients are reporting on their home living situation, not their stay in residence. Thus, it can be postulated that certain family factors (e.g., level of family complexity) that are known to influence QoL may contribute to a child/youth requiring inpatient as opposed to outpatient services. This is consistent with a study conducted by Stewart and colleagues (65) that found youth were more likely to be readmitted to inpatient psychiatry if they had a dysfunctional relationship with their family members.

As predicted, depressive symptoms were significantly associated with the greatest number of QoL sub-domains. Specifically, higher depressive symptoms and anhedonia were associated with lower social QoL, and higher depressive symptoms alone were associated with lower individual and basic needs QoL; no mental state indicators were predictive of QoL pertaining to services

This suggests that across all outcome variables, depressive symptoms and anhedonia were the only mental state indicators predictive of a lower QoL, which both fall under the umbrella of internalizing symptoms. Weitkamp et al. (66) similarly found that internalizing, as opposed to externalizing symptoms, predicted lower self-reported QoL. In particular, they found that there was a relationship between higher internalizing symptoms and lower QoL within the psychological and social sub-domains. This supports the present study's findings (as their psychological domain may be likened to our individual domain), with depressive symptoms predicting lower individual QoL and anhedonia and depressive symptoms predicting lower social QoL. Taken altogether, the results of both the present study and prior research (66) indicate that certain mental state indicators have an effect on particular QoL domains, but not a generalized effect across all QoL domains. This underscores the importance of continuing to consider various sub-domains when researching QoL, in order to differentiate the impact of certain determinants on specific areas of QoL.

As previously noted, depressive symptoms was the only predictor of lower QoL on the individual sub-domain. While different QoL measures have varying sub-domains, there is some overlap between the emotional functioning sub-domain of other QoL tools and the individual sub-domain of the interRAI QoL-ChYMH (which includes items such as “I feel good about myself”) (13). Bastiaansen and colleagues (67) also found higher depressive symptoms were related to lower emotional functioning. Depressive symptoms were also the only predictor of lower QoL on the basic needs sub-domain, representing a decrease in satisfaction with, for example, safety and food. Similarly, others have found that children from families in disadvantaged social classes had slightly lower HR-QOL compared to children from families in more advantaged social ones (68).

While depressive symptoms were significantly correlated to QoL at both the bivariate and multivariate level, anxiety symptoms were only associated with QoL at the bivariate level. Hence, when the relationship between anxiety and QoL is examined singularly, a significant relationship exists; however, this finding does not hold true when other clinical variables are entered into the analytic model. Similarly, Freire and Ferreira (69) examined the impact of various clinical factors, including stress, anxiety symptoms, and depressive symptoms, on domain-specific (i.e., psychological, physical, social, autonomy, and school) QoL in youth. Similar to the findings reported herein, depressive symptoms predicted the greatest number of QoL sub-domains, and anxiety symptoms did not predict domain-specific QoL in this multivariate model.

While depressive symptoms were also associated with social QoL at the multivariate level, anhedonia was the strongest predictor of this particular QoL domain. Interestingly, Barge-Schaapveld et al. (70) found that even after controlling for physical complaints, mood, and enjoyment of activities, depression continued to have a significant effect on QoL thereby indicating that other unmeasured aspects of depression have an effect on self-reported well-being, such as those related to anhedonia. Overall, the independent effects of anhedonia and mood-related depressive symptoms on QoL sub-domains were able to be teased apart in the present study because they were both included as distinct predictors in the same multivariate model.

Positive symptoms were not a significant predictor of QoL. Previous research conducted on patients with schizophrenia also found that negative, but not positive, symptoms are associated with HR-QOL and subjective QoL (71). Moreover, among individuals who are vulnerable to psychosis, negative symptoms are strongly associated with QoL and functioning ability; however, the same is not true for positive symptoms (72).

Finally, similar to anxiety and positive symptoms, the present study did not find a significant association between hyperactivity/ distractibility and domain-specific QoL. Previous research (20) found ADHD to be associated with QoL when parental reports were used to measure QoL. However, there is a much weaker, and at times, non-existent relationship when the child or youth reports on their own QoL. This discordance may be due to the fact that children with ADHD may have an overly optimistic view of their situation, wish to conceal their problems, or rush through the survey due to their impulsive nature (20, 73). Moreover, some explanations for the discordance between parent-proxy and child self-reports of QoL in ADHD populations focus more so on the role of the parent. For example, parent bias could conflate the results (74). Studies have found that parents of children with ADHD tend to report increased marital conflict, parenting stress, depression, and alcohol consumption, potentially influencing perceptions of their own QoL that has been negatively impacted by their child's disorder (75–77). Consequently, future research needs to use self-report QoL measures in order to gain the perspective of the children and adolescents themselves.

### Clinical Implications

Because of their significant clinical utility, interRAI QoL measures are increasingly being used within healthcare, including mental health research. On a broad level, interRAI QoL data can inform resource allocation within various health service sectors. On a more individual level, QoL measures can be used to monitor a patient's progression over time and assess the effectiveness of a particular intervention, or provide assistance in the diagnostic process by providing a window into the child or youth's perspective. This allows the clinician to gain insight into the areas of functioning the child or youth is struggling with most, which can subsequently inform care planning.

Anhedonia and depressive symptoms are two core symptoms of major depressive disorder (MDD), which has been described as a major public health problem due to its many negative effects, including disability, secondary morbidity, and high mortality (78). MDD is also correlated with a high economic cost, with annual direct and indirect costs totaling $43 billion (79). Taken altogether, these results point to the potential advantage of regularly screening for depressive and anhedonia symptoms in both preventative work and treatment planning, which could potentially improve individual QoL while also reducing the disorder's economic burden. Overall, domain-specific research can be utilized to achieve a better understanding of which domains of QoL are affected by specific psychopathologies, thereby allowing clinicians the ability to focus on those particular QoL domains during assessment, diagnosis, and treatment planning.

### Limitations

While the current study has several notable strengths, including internationally-used assessment instruments, it also has a few limitations. First, due to the fact that both assessment tools (the ChYMH and QoL-ChYMH) were completed at a tertiary facility in the province of Ontario, participants of the study represent a convenience sample and were not randomly selected. Second, the findings may not be generalizable to a community-based sample since all of the children and youth assessed were receiving inpatient or outpatient mental health care from a tertiary facility. Finally, because of the cross-sectional nature of the data, the study is unable to draw any conclusions about causality and, consequently, the directionality of the findings cannot be determined.

### Future Directions

There are many interesting avenues for future research that can build upon the present study. For example, one potential area for further investigation is to examine whether the current study's findings are generalizable to a community-based sample. Furthermore, longitudinal studies can be conducted to explore whether there are critical periods during which certain mental state indicators are more predictive of lower QoL; for example, depressive symptoms may be more predictive of QoL in adolescence compared to childhood. There is an important need to develop new knowledge regarding the mitigating factors impacting QoL during critical developmental periods as well as across the lifespan.

## Data Availability Statement

The original contributions presented in the study are included in the article/supplementary material, further inquiries can be directed to the corresponding authors.

## Ethics Statement

The studies involving human participants were reviewed and approved by Western University's ethics review board (REB #106415, #112885). Written informed consent from the participants' legal guardian/next of kin was not required to participate in this study in accordance with the national legislation and the institutional requirements.

## Author Contributions

All authors contributed to the formulation of the ideas presented in the study. AC and NL performed the statistical analysis. All authors were involved in the writing and reviewing of the final manuscript.

## Conflict of Interest

The authors declare that the research was conducted in the absence of any commercial or financial relationships that could be construed as a potential conflict of interest. The Handling Editor JF declared a past co-authorship/collaboration with one of the authors SS.

## Publisher's Note

All claims expressed in this article are solely those of the authors and do not necessarily represent those of their affiliated organizations, or those of the publisher, the editors and the reviewers. Any product that may be evaluated in this article, or claim that may be made by its manufacturer, is not guaranteed or endorsed by the publisher.
